# Education or Provision? A Comparison of Two School-Based Fruit and Vegetable Nutrition Education Programs in the Netherlands

**DOI:** 10.3390/nu12113280

**Published:** 2020-10-26

**Authors:** Angeliek Verdonschot, Emely de Vet, Jolien van Rossum, Anouk Mesch, Clare E. Collins, Tamara Bucher, Annemien Haveman-Nies

**Affiliations:** 1 Consumption and Healthy Lifestyles, Wageningen University and Research, 6700 EW Wageningen, The Netherlands; emely.devet@wur.nl (E.d.V.); jolien38vanrossum@hotmail.com (J.v.R.); anouk.mesch@wur.nl (A.M.); annemien.haveman@wur.nl (A.H.-N.); 2 Priority Research Center for Physical Activity and Nutrition (PRCPAN), The University of Newcastle, Callaghan, NSW 2308, Australia; clare.collins@newcastle.edu.au (C.E.C.); tamara.bucher@newcastle.edu.au (T.B.); 3 School of Health Sciences, Faculty of Health and Medicine, The University of Newcastle, Callaghan, NSW 2308, Australia; 4 School of Environmental and Life Sciences (SELS), Faculty of Science, The University of Newcastle, Callaghan, NSW 2308, Australia

**Keywords:** nutrition education, FV provision, primary school children, nutrition knowledge

## Abstract

A healthy diet is important for optimal child growth and development. School-based opportunities to encourage children to achieve healthy eating behaviors should be explored. Nutrition education programs can provide school children with classroom-based nutrition education and access to fruits and vegetables (FV). However, the effectiveness of specific program components implemented separately has not yet been comprehensively evaluated. The current study examined effectiveness of individual components of two programs targeting primary school children (*n* = 1460, *n* = 37 schools) aged 7–12 years. Nutrition knowledge and FV consumption were measured using a student questionnaire, and presence of school food policies was measured in the teachers’ questionnaire. A quasi-experimental design with three arms compared: (1) schools that implemented both programs: FV provision + education (*n* = 15), (2) schools that implemented the FV provision program only (*n* = 12), (3) schools that did not implement either program (*n* = 10). Outcomes were assessed pre-intervention (T0), during the intervention (T1), and 6 months post-intervention (T2). Results indicated a significant increase in nutrition knowledge for children attending schools that had participated in both programs, compared to control schools (*p* < 0.01), but no significant increase in FV intake. In schools without food policies, FV provision alone contributed to an increase in child FV intake (*p* < 0.05).

## 1. Introduction

Consuming adequate amounts of fruits and vegetables (FV) as part of the healthy diet could help prevent non-communicable condition, including obesity, type II diabetes and cardiovascular disease [1]. These types of diseases are highly prevalent in high income countries, and on the rise in low- and middle-income countries [2,3]. This trend is alarming since it has a major impact on modern societies, both economically and socially [4]. Since adult eating behavior develops from an early age and schools are an effective learning environment where child eating behaviors could be targeted, school-based nutrition education programs could potentially have impact on population health [5].

In the last decade, several school-based nutrition education programs have been developed and evaluated. Tak et al. (2009) indicated a significant increase in FV intake and nutrition knowledge in children, as a result of the Dutch nutrition education program ‘Schoolgruiten Project’, which focused on the provision of FV (environmental component) [6]. The ‘5 a day’ program in Italy was based on a curriculum approach including lessons and educational videogames (educational component), found an increase in children’s FV consumption [7]. In addition to single component interventions, programs with a multi-component approach have also been implemented, with a significant effect on FV consumption. In Canada, the program ‘Action Schools! BC—Healthy Eating’ that used a multi-component approach (with educational (lessons and tasting activities), environmental and family components) increased children’s FV intake significantly (+0.18 serving), compared to the control group (−0.79 serving, *p* ≤ 0.05) [8]. Similar results were found in another multi-component program, where children in the experimental group consumed more FV (F: 29 g and V: 6 g, *p* ≤ 0.01) compared with children in the control group [9]. While several programs have been shown to be effective, other comparable evaluation studies found little to no effects [10,11,12,13,14].

Program effectiveness depends on several factors, including program content, aims, methods, activities and type of approach [15]. A literature review by Mak et al. (2016) grouped these highly varied programs into three categories: (1) education component programs (e.g., classroom-based learning, experiential learning, games/competitions (rewards and incentives) and behavior change approaches), (2) environmental component programs (e.g., the availability of FV and education of school staff) and (3) parental/family component programs (e.g., homework with children, parent involvement in activities in school) [16]. This review included 66 successful intervention studies that reported a significant increase in children’s FV intake, with 16 studies including education components only and 50 studies using multi-component approaches (education, environment and/or parental).

Current evidence indicates that interventions implementing a multi-component approach seem to be more effective, compared to interventions adopting a single component approach [16,17,18]. However, it is often unclear which individual component contributes to the measured intervention effect [15,16,19]. Secondly, the heterogeneity among outcomes measures and methods used makes program and component comparisons more complex. Consequently, further insight is required in regard to effectiveness of individual components of school-based nutrition education programs.

In addition to the school-based programs, school policies related to FV consumption at school may be relevant in supporting the program success. A review, conducted by Micha et al. (2018), indicated that direct FV provision policies increased fruit intake on average by 0.27 servings per day and vegetable intake by 0.04 servings per day, according to 26 studies (15 studies on fruit and 11 on vegetables). Other effective food policies were related to school meal standards, including policies on school meal (mainly lunch) standards generally targeted FV, dietary fats and sodium. Considering the FV school meals standards, some multi-component studies found a significant increase in children’s fruit intake, and a non-significant increase in vegetable intake [20,21]. Yet, up to present, most evaluation studies of nutrition education programs do not report the school food policy context.

In the Netherlands, two widely implemented school-based national nutrition education programs are EU-Schoolfruit and Taste Lessons (in Dutch: ‘Smaaklessen’) [22]. EU-Schoolfruit includes an environmental component and provision of FV in participating schools. In addition, EU-Schoolfruit offers one nutrition lesson that can be implemented by the teacher for each grade of primary school (grades 1–8, children aged 4–12). Taste Lessons is based on an educational component which consists of five lessons for each grade, discussing various topics in relation to five themes: ‘taste’, ‘nutrition and health’, ‘cooking’, ‘food production’ and ‘consumer skills’. Lessons can be implemented by teachers across the whole school year. Regarding Dutch school food policies, most primary schools indicate they have a written food policy that indicates what is allowed to be brought to school, although policies are not enforced [23].

The current study aim was to evaluate the effect of FV provision alone and combined with nutrition education on FV intake and nutrition knowledge in school children aged 7–12 years old. A secondary aim was to stratify results by presence or absence of school food policies.

## 2. Materials and Methods

### 2.1. Intervention

Intervention effectiveness was compared for two Dutch nutrition education programs that each have a different focus. EU-Schoolfruit focuses on FV provision and Taste Lessons focuses on nutrition education.

EU-Schoolfruit is a Dutch nationwide nutrition education program for primary schools, developed in 2009 and financed by the European Union [24]. Participating primary schools receive three pieces of FV per child per week for a period of 20 weeks (November–April) in order to promote FV consumption. Every year, around 3000 Dutch primary schools, out of a total approximate amount of 7000, participate voluntary in this program.

Taste Lessons, developed in 2006, is another Dutch national school-based nutrition education program for primary schools [25,26]. The program consists of five lessons for each grade, discussing various topics in relation to five themes: ‘taste’, ‘nutrition and health’, ‘cooking’, ‘food production’ and ‘consumer skills’. Each lesson consists of several activities including experiments, cooking and tasting. Some lessons include home assignments for children to complete with their parents. Additionally, tips for extra activities, such as visiting a farmer, are provided within the program. Teachers can implement Taste Lessons that best fit their schedule over the whole school year. On average, 5000 Dutch primary schools implemented the Taste Lessons program in the period from January 2017–June 2020.

### 2.2. Study Sample and Recruitment

The current study included three study groups: (1) schools that implemented EU-Schoolfruit and Taste Lessons, the ‘FV provision + Education (FV + Ed) group’, (2) schools that implemented only EU-Schoolfruit, the ‘FV provision (FV) group’, and (3) schools that did not implement either program, the ‘Control group’. As the Education program has already been evaluated in previous studies [25,27,28], but evaluation studies have not been conducted on the FV provision program, the current study sought to measure the effect of the FV provision program, with and without an education component, as we were interested in the multi-component approach.

Primary schools throughout the Netherlands were invited to participate in the ‘FV + Ed group’ or ‘FV group’ of current study through an advertisement on the EU-Schoolfruit webpage, in the EU-Schoolfruit newsletter, on the Taste Lessons webpage, and through Healthy School Advisors (of the Dutch Municipal Health Services). Additionally, Dutch Municipal Health Services recommended schools that could be approached for participation. Schools that had the intention to implement EU-Schoolfruit and Taste Lessons in school year 2018/2019 were placed in the FV+Ed group. Schools that had the intention to only implement EU-Schoolfruit, and had in the previous two years (school year ‘16/’17 and ‘17/’18) no experience in Taste Lessons, were placed in the FV group. Schools could not be randomly assigned to an intervention group since experience in Taste Lessons would bias results, with child nutrition knowledge likely higher due to prior participation in Taste Lessons. In addition, EU-Schoolfruit is a whole school program and participation could not be dictated by the current study.

**Figure 1:**
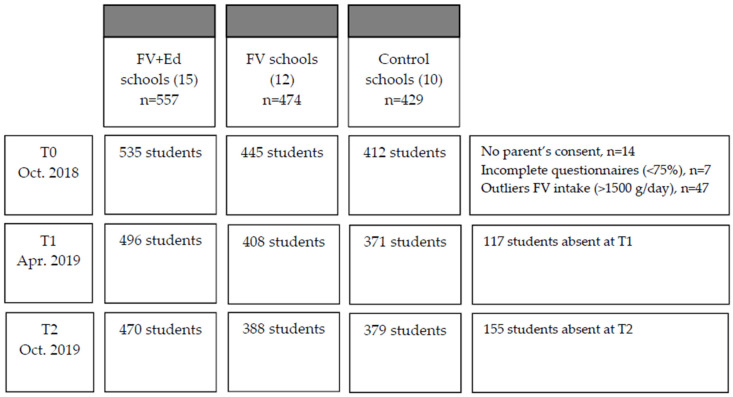
Study sample during the measurements and analyses (*n =* number of students).

To recruit schools for the control group, the Dutch Municipal Health Services again recommended suitable schools that could be approached and that met the control group criteria (no experience in either the FV provision program via EU-Schoolfruit or the education program via Taste Lessons and did not implement any nutrition education program in school year 2018/2019). Furthermore, a public list of all Dutch primary schools was used to randomly contact schools by phone to invite them to participate in the study [29]. From this public list, schools that implemented EU-Schoolfruit or Taste Lessons in the last two years (school year ‘16/’17 and ‘17/’18), or schools that intended to participate in another nutrition program in 2018/2019 were excluded. The recruitment resulted in 37 schools and 1460 children from grade 6 and 7 (Figure 1).

### 2.3. Study Design and Procedure

To assess the effect of FV provision and education a quasi-experimental design was used including three arms: (1) the FV + Ed group (schools, *n* = 15), (2) the FV group (schools, *n* = 12) and (3) the control group (schools, *n* = 10). Outcomes were assessed pre-intervention (baseline, T0), during the intervention (approximately 6 months after baseline, T1), and 6 months’ post-intervention (approximately 12 months after baseline, T2).

Before data collection, a pilot study using the child questionnaire was conducted, in two classes (combined grade 6 and 7) from two different schools in Wageningen (The Netherlands). Following this pilot, illustrations were added to the questionnaire to make it more attractive and improve comprehension by the children.

In the starting phase of the 2018–2019 school year (T0), research assistants visited participating schools to collect baseline information. The children from grades 6 to 7 were asked to complete a 30-item-questionnaire in the classroom under the supervision of a research assistant. After the start of the FV provision program (EU-Schoolfruit) (November 2018), the teachers from the FV + Ed schools were asked to implement five lessons from the education program (Taste Lessons), within the 20 week period they implemented the FV provision program (November 2018–April 2019). In the last couple of weeks of the FV provision program (April 2019), the second measurement (T1) was conducted, with children completing the same questionnaire as baseline (T0). The third follow-up measurement (T2) was conducted six months after the FV provision program had finished. The study was approved by the Social Science Ethical Committee (SSEC) from Wageningen University and Research and was pre-registered in the Netherlands Trial Register (ID: NL7317).

The three measurements (child questionnaire) in the control schools took place in the same period as the FV + Ed and FV groups. The effect of FV provision and education was measured by comparing changes between the different times (T0, T1 and T2) in nutrition knowledge and FV consumption between the three groups (FV + Ed, FV and control). Questionnaire items about the implemented food policy in schools were added to a 15-min-questionnaire for the teachers (*n* = 61) of participating classes in the second measurement (T1).

### 2.4. Measures

#### 2.4.1. Outcome Variables

##### Nutrition Knowledge

Children’s nutrition knowledge was assessed by 24 questions related to what the children were taught during the education program (Taste Lessons) (Table 1). Questions were based on previous research about the effectiveness of nutrition education [27,30]. Additionally, the response options from the previous questionnaire by Vereecken et al. (2012) were supplemented with an ‘I don’t know’ option. Correct answers received a score of 1, and incorrect and ‘I don’t know’ answers scored a 0.

The total score for each component (cluster) was divided by the number of questions answered to calculate the mean score per component. To calculate the total nutrition knowledge score, the mean scores of all components (clusters) were summed.

##### Fruit and Vegetable Consumption

Children’s fruit and vegetable (FV) intake was measured using a validated 24 h recall as described elsewhere [31]. Briefly, the 24 h recall recorded FV consumption for the previous (school) day and was collected on Tuesday to Friday as a class in school time, administered by researchers. As suggested by Haraldsdóttir et al. (2005), the 24 h recall consisted of three time intervals: (1) the morning (breakfast and morning snack), (2) the afternoon (lunch and afternoon snack), and (3) the evening (dinner and evening snack). Each time interval started with two general questions such as ‘Did you eat something during breakfast or in the school break yesterday morning?’ and ‘Did you eat fruit or vegetables during breakfast or in the school break yesterday morning?’. These questions aimed at making the children think of their actual intake of the previous day. After that, the students were asked to fill in a pre-coded table specifying the type and amount of FV eaten during three time intervals (Table 2). Images of a 0.5 and 1.0 L water bottle and the type of serving spoon were listed in the questionnaire as prompts for portion sizes. If their eaten FV were not listed, they could enter these in the open space that was provided in the table. Similar to Haraldsdóttir et al. (2005), legumes, nuts, juices, smoothies and potatoes (except sweet potato) were not included. To convert the reported portion sizes into grams, Dutch standard portion sizes were used [32]. If the type and amount of FV was not mentioned or unclear, the most commonly type eaten and average amount was reported, based on the Dutch National Food Consumption Survey [33]. The NEVO online recipes database (in Dutch: ‘Nederlands Voedingsstoffenbestand’) was used to convert vegetable percentages of soups and mixed dishes into grams (RIVM, 2016).

#### 2.4.2. Personal Characteristics

Characteristics of the children and teachers were measured. The child questionnaire contained items about their age (in years), sex and grade (6, 7 or 8), whereas the teacher questionnaire included items about their age (in years), sex and teacher experience (in years).

#### 2.4.3. Contextual Factors

##### School Characteristics

Characteristics of the schools were measured with a questionnaire for the teachers, containing items about the size of the school and the principle (public versus special). In addition, information about the social economic position (SEP) of the neighborhood of the school was obtained from a Dutch online database with values from −3.4 (high SEP) to 5.2 (low SEP), with a mean score of 0 [34]. These scores were based on degree of education, income, and work status of households within postal code districts.

##### School Food Policy

The questionnaire for teachers at T1 contained four items about the school’s rules and policies implemented related to FV consumption (Table 1). Response options regarding type and content were grouped together to create a new variable on food policy, including three categories: (1) no FV policy, (2) morning FV rule, and (3) morning FV rule + extra FV policy. The option ‘no FV policy’ indicated that the school did not implement morning FV rules. The option ‘morning FV rule’ indicated the children ate a healthy snack during the morning break (e.g., fruits, vegetables, or a wholegrain sandwich). The option ‘morning FV rule + extra FV policy’ indicated the schools implemented on top of the morning FV rule another FV policy, such as ‘healthy birthday treat policy’ or ‘healthy lunch policy’. Healthy birthday treat policy means that the school requests the guardians to keep the birthday treats small and not high in calories (e.g., by using FV), or to replace the treat with a small non-food item. Healthy lunch means that the school request guardians not to put any unhealthy foods in their children’s lunchboxes [35]. When teachers from the same school reported different active FV rules or food policies, the teachers were requested for clarification or the school website was explored.

### 2.5. Statistical Analysis

First, equality across the study groups was tested via the Kruskal–Wallis, followed by post hoc test; the Dunn test (continuous variables) and the Chi-square (categorical variables) tests. Based on these tests, the study groups were comparable for the variables age, sex, grade and FV intake, but not for the variable nutrition knowledge, whereby the control group had a significant higher level of nutrition knowledge, compared to the other two groups (FV + Ed and FV) (*p* < 0.05). Subsequently, demographic characteristics of the children, teachers and schools were evaluated based on means and standard deviations from the continuous variables and frequencies from categorical variables for every condition (FV + Ed, FV and control). Multilevel regression analyses were conducted to measure the effect of FV provision and education on children’s nutrition knowledge and FV intake including three levels: (1) student (2) class and (3) school. To evaluate change in children’s FV intake and nutrition knowledge in short- and long-term, results from baseline (T0) were compared with the second (T1) and third measurement (T2). Next, to assess the association between the actual amount of lessons (via Taste Lessons) implemented and the change in nutrition knowledge, a multilevel analysis was conducted, with implementation dose (number of lessons that the children received) as independent variable and change in knowledge as dependent variables. For this analysis, the implementation dose was split into two categories: low amount (<3 lessons) and high amount (>3 lessons). Following, to evaluate the impact of FV rules and policies on the effect of FV provision and education, food policy was added to the model as moderator. Subsequently, the multilevel regression analyses were stratified across levels of food policy. A *p*-value of less than 0.05 was considered to be significant. Linearity as well as normality and homogeneity of residuals were checked, whereby modest deviations from normality and homogeneity were observed. All multilevel analyses were adjusted for age and sex, to account for confounding. The SEP score and status of school food policies were non-significant confounders and therefore not included in the analysis. All analyses were performed using the software R, version 3.6.1 [36].

## 3. Results

### 3.1. Demographic Characteristics

At baseline, the mean age of all participating children was 9.6 (standard deviation (SD):0.7) years and did not differ between groups (*p* = 0.109). Both sex and school grade were equally represented in all groups (*p* = 0.572 and *p* = 0.494 respectively). Children’s nutrition knowledge was significantly higher in the control group (mean: 3.2, SD: 0.8), compared with the FV + Ed- and FV group (mean: 2.9, SD: 0.8 and mean: 2.9, SD: 0.8, *p* < 0.05). Total FV intake at baseline was found to be not significantly different across study groups (*p* = 0.856), with a mean of 330 (SD: 265) grams per day. The mean age of the teachers was 40 years (SD: 12), with all groups including more female than male teachers (80.3% F and 19.7% M). The mean experience level as a teacher was 17 years. The control group had a relatively high school neighborhood SEP (−0.38, SD: 0.63), compared to the other groups (FV 0.34, SD: 0.91 and FV + Ed 0.54, SD: 0.91). More intervention schools had implemented a policy, compared to control schools (Table 3).

### 3.2. Effect on Children’s Nutrition Knowledge

In schools that implemented both programs (FV + Ed), a significant increase in children’s nutrition knowledge was identified, in both short- (T1) and long term (T2) compared to the control group (*p* < 0.01 and *p* < 0.05 respectively) (Table 4). In addition, based on results from the FV + Ed group, the change in nutrition knowledge was significantly higher when 3–5 lessons were conducted, compared to conducting ≤2 lessons, in short- (T1) and long term (T2) (β = 0.18; 95%CI:0.03, 0.33, *p* = 0.016 and β = 0.23; 95%CI:0.08, 0.38, *p* = 0.003 respectively). FV provision alone did not increase children’s nutrition knowledge.

### 3.3. Effect on Children’s FV Intake

In both intervention schools (FV and FV + Ed), no significant difference in children’s FV intake was identified in either the short- or long-term, compared to the control group (FV *p* = 0.293 and *p* = 0.179; FV + Ed *p* = 0.104 and *p* = 0.808 respectively) (Table 5). Results demonstrated a non-significant increase in FV intake during the intervention (T1) (FV 22 g/day/student and FV + Ed 35 g/day/student), compared to the control group (−7 g/day/student). In addition, non-significant results were found based on the follow-up measurement (T2), whereas an increase in FV intake was identified in schools that implemented EU-Schoolfruit only (2 g), and children’s FV intake decreased in the FV + Ed group and control group (FV + Ed −34 g/day/student and control −40 g/day/student).

### 3.4. Schools Stratified by School Food Policy Status

**Figure 2:**
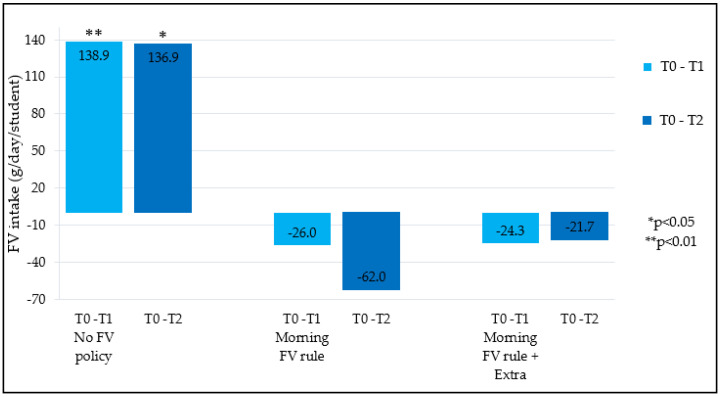

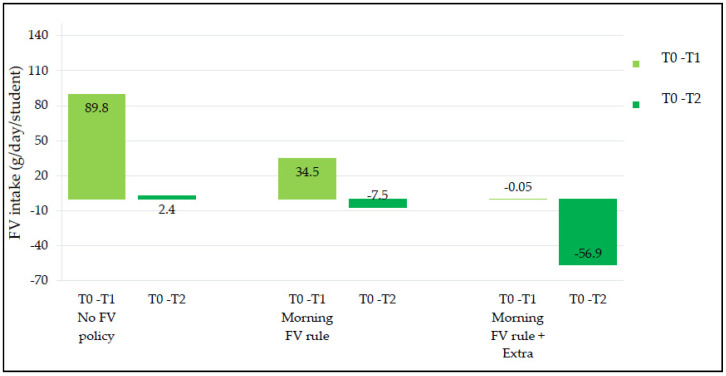
Difference in children’s FV intake stratified by school food policy in FV group, compared to control group; Difference in children’s FV intake stratified by school food policy in FV + Ed group, compared to control group.

Changes in FV intake between the three time periods (T0, T1 and T2) were greatest in intervention schools that did not have a food policy (Figure 2). Results indicated a significant increase in FV intake, in both the short- and long-term, in FV schools without a food policy, compared to the control schools (*p* < 0.01 and *p* < 0.05 respectively). In schools with a food policy, change in children’s nutrition knowledge was not significantly different, compared to schools without a food policy.

## 4. Discussion

### 4.1. Main Results

The current study aimed to evaluate the effect of FV provision alone and combined with nutrition education on FV intake and nutrition knowledge in primary school children, in schools stratified by food policy status. Results indicated that nutrition knowledge significantly increased in children who received both the programs including FV provision and nutrition education, compared to school children who did not receive nutrition education (control group). This increase in nutrition knowledge remained significant six months post intervention. However, FV provision and nutrition education had no direct effect on children’s FV intake. In the subgroup analysis based on stratification by presence or absence of a school food policy, in schools without food policy a significant effect of a FV provision program was found on children’s FV intake, compared to the control group.

### 4.2. Effect on Children’s Nutrition Knowledge

In line with other studies [37,38,39,40] the results of the current study indicate that receiving education led to a significant increase in nutrition knowledge in children. This increase in knowledge in children remained significant in the long-term and is in line with previous research, which identified a significant increase in children’s nutrition knowledge following education [28]. Additionally, the change in nutrition knowledge was greater in children who received more educational lessons, compared to children who received two or less lessons. This effect was observed in both the short- and long-term. Despites only 2.9 lessons, out of the 5 total offered lessons being implemented, the educational program had a significant positive impact. It could be expected that the effect on children’s nutrition knowledge would be the greatest following implementation of all lessons in the FV provision + education group.

In the current study, the classroom-materials used in the original version of EU-Schoolfruit program were omitted in order to examine the effect of FV distribution and the education component separately. As expected, the EU-Schoolfruit program in the current study, which included FV distribution only and no classroom component, did not impact children’s nutrition knowledge. However, the combination of EU-Schoolfruit and Taste Lessons did demonstrate an increase in children’s nutrition knowledge and this change in knowledge is therefore attributed to the education component of Taste Lessons.

### 4.3. Effect on Children’s FV Intake

No significant effect on children’s FV intake was found for either FV provision program alone or combined with the educational program. Results from the 24 h recall data indicate an increase of 29.7 g (FV group) and 43.2 g (FV + Ed group) in FV intake, but this was not statistically significant. These findings are in line with results of a systematic review that found a mean post-intervention daily increase of 20–30 g FV intake [17]. The non-significant result in the current study may be explained by the use of the 24 h recall method. This method limited the possibility to take into account day-to-day variation and large variations in FV intake were found, resulting in wide confidence intervals. In addition, 24 h recalls rely largely on memory and cognition, potentially influencing the accuracy of child-reported intakes. More precise measurement methods would likely lead to more precise FV intake estimates, but also to a higher participant burden, higher costs, and likely lower participation rates.

### 4.4. Schools Stratified by School Food Policy Status

Results suggested that whether or not school food policy is implemented may influence the potential for the FV provision program (EU-Schoolfruit) to affect children’s FV intake. A significant increase in children’s FV intake was found in both short- and long-term but only in schools without food policy. In schools with food policies no significant effects were found. This is in contrast with previous research, that suggested the effectiveness of such programs will increase if school food policies are added [25,41]. This could be explained by the fact that the FV delivered by EU-Schoolfruit might potentially replace the FV that would be taken to school if there was no EU-Schoolfruit. Therefore, FV from EU-Schoolfruit (mostly eaten in the morning breaks), might not change the amount of eaten FV. Vice versa, in schools without food policies, the FV delivered by EU-Schoolfruit could replace other snacks, potentially resulting in increased FV intake. In these schools a food policy could be used to encourage parents to give FV to their children instead of other snacks after the period of the EU-Schoolfruit program. In schools in the Netherlands, food is usually brought to school by the children themselves. It is therefore expected that the effectiveness of a food policy is dependent on the implementation and communication to families in regard to adherence to the food policies by the children and/or parents [23]. However, this explanation was not examined in current study and needs further research. Moreover, the school food policy should fit with the school’s needs and therefore more insight is needed into motives of schools that do not have school food policies.

### 4.5. Comparison of Different Components

Based on a systematic review including 29 school-based programs, multi-component programs (*n* = 16) tended to result in larger improvements in FV intake (varying from −0.23 to +1.7 FV portions, compared to control group), compared to single-component programs (*n* = 13) (varying from 0.0 to + 1.9 FV portions, compared to control group) [17]. In contrast, the current results identified that this multi-component program (using an environmental- and educational component—FV + Ed group) was not more effective, compared to other single component programs (FV group). Further, implementing school food policies also did not improve the effectiveness of nutrition education programs.

### 4.6. Strengths and Limitations

The current study was conducted in a large sample of 37 primary schools including 1392 children throughout the Netherlands. The interventions (EU-Schoolfruit and Taste Lessons) were implemented in primary schools, which contributed to the external validity. Further, previously validated methods were used to measure FV intake (24 h recalls [31]) and nutrition knowledge in children [27,30]. The self-reported methods were chosen since this was most suited for collect data from many children at the same time. However, a limitation of the 24 h recall was that several children experienced difficulties in recalling their FV intake for the previous day. During the measurements, some children either had difficulty remembering what they had eaten or found it hard to estimate their portion sizes. Other methods that can be implemented to measure FV intake in children include weighed records or to conduct the 24 h recall orally by phone or face to face [42].

A quasi-experimental design was used, including control schools and a baseline measurement (T0). This made it likely that any effect on knowledge and FV intake can be explained by EU-Schoolfruit and/or Taste Lessons. However, participating schools were not randomly assigned to the intervention (FV/FV + Ed) or control group, since the control group was recruited differently than the intervention group. The different recruitment may have impacted the results since intervention schools may be more active in encouraging healthy eating in school via programs compared to control schools based on their experience. However, baseline results did not support this hypothesis, indicating children from control schools had higher nutrition knowledge, compared to children from intervention schools. This may be explained by the potential difference in social economic position (SEP) indicated between control and intervention schools. The SEP score was based on degree of education, income, and work status of households within postal code districts and may be more related to nutrition knowledge. Nevertheless, we adjusted for this difference in nutrition knowledge at baseline in our analyses and it did not influence the results. In addition, all participating schools were comparable based on age, grades (6 and 7) and sex. Only little differences in food policies, principle, sizes, and location of the schools were observed. The current study included three measurements (T0, T1 and T2), over a period of one year, with program effectiveness in the longer term (>12 months) not examined. Therefore, it is recommended that future research evaluates program effectiveness on long term (>12 months) outcomes, although effects were already limited in the first year.

## 5. Conclusions

The current study found a significant increase in children’s nutrition knowledge as a result of participating in both a FV provision program and an education program and highlights the importance of policy context. To improve future evaluations of school-based health promoting programs, future studies should be conducted within the school food policy context, with more accurate quantification of FV intake.

## Figures and Tables

**Table 1:** Items used to measure nutrition knowledge and FV intake (children) and food policies in school (teachers)**.**

Outcome Measure (Children/Teachers)	Theme (*n =* Items)	Example Question	Answer Options (^#^ = Correct Answer)
Nutrition knowledge (children)	Healthy food choices (5)	‘What is most healthy to snack?’ *(images of the products)*	(1) Chips (2) M&M’s (3) Popcorn ^#^ (4) I don’t know
	Recommended portions (6)	‘How much vegetable do you (aged 8-11) need every day to grow and stay healthy according to The Wheel of Five (in Dutch: ‘De Schijf van Vijf’)?’	(1) 0–50 g (2) 50–100 g (3) 100–200 g ^#^ (4) 200–300 g (5) 300–350 g (6) I don’t know
	The Wheel of Five (in Dutch: ‘De Schijf van Vijf’) (3)	‘Which food product does not belong in the food group according to The Wheel of Five?’ *(images of the products)*	(1) Pinto beans ^#^ (2) Banana (3) Tomato (4) Plum (5) I don’t know
	Nutrient content (5)	‘Whole grain bread contains….’ (*circle the correct answer*)	(1) Less vitamins and minerals than white bread (2) As much minerals and vitamins as white bread (3) More vitamins and minerals than white bread ^#^ (4) I don’t know
	Senses (3)	‘You can taste with your tongue if there is any salt in the food/drink you are tasting’ *(is this statement true or false?)*	(1) True ^#^ (2) False (3) I don’t know
	Food production (2)	‘Organic products contain similar pesticides as conventional products’ *(is this statement true or false?)*	(1) True (2) False ^#^ (3) I don’t know
FV intake (children)	FV intake at previous school day (6)	‘What type of vegetable/fruit, and how much did you eat yesterday?’	Precoded table (see Table 2)
School food policy (teachers)	Type and content (4)	‘Does your school have an active food policy?’ *(multiple answers possible)*	(1) Yes, with regard to healthy snacks during the mid-morning break (2) Yes, with regard to healthy lunch (3) Yes, with regard to healthy drinks (4) Yes, with regard to healthy birthday treats (5) Yes, with regard to other, namely… (6) No

**Table 2:** One of the precoded questions on FV intake in the 24 h recall.

Did you Eat Fruit or Vegetables Yesterday Morning? (Write Yes/No)					
If Yes, What Kind of Fruit or Vegetable and How Much? (Write 1 if You Ate One Apple, Write Half if You Ate Half an Apple. If Your Fruit or Vegetable is not Listed below, You Can Fill it in the Empty Rows Below)					
Fruits in the Morning			Vegetables in the Morning		
Apple	…..	Piece	Cucumber	…..	Slides
Banana	…..	Piece	Cherry tomatoes	…..	Pieces
Mandarin	…..	Piece	Capsicum	…..	Strips
Grapes	…..	Hand	Carrot	…..	Piece
….	…..	…..	…..	…..	…..
….	…..	…..	…..	…..	…..

**Table 3:** Descriptive statistics of the children, teachers, and schools.

	Control	FV	FV + Ed
Children (*n* = 1392)	*n* = 412	*n* = 445	*n* = 535
Age (years), mean (SD)	9.6 (0.7)	9.5 (0.7)	9.6 (0.7)
Sex, *n* (%)			
Boy	203 (49.4)	212 (47.6)	273 (51.0)
Grade, *n* (%)			
Grade 6	204 (49.5)	236 (53.0)	266 (49.7)
Grade 7	208 (50.5)	209 (47.0)	269 (50.3)
Nutrition knowledge * T0, mean (SD)	3.15 (0.79)	2.92 (0.82)	2.92 (0.81)
Total FV intake (gram) T0, mean (SD)	326 (266)	339 (277)	326 (255)
Teachers (T1) (*n* = 61)	*n* = 16	*n* = 23	*n* = 22
Age (years), mean (SD)	40.1 (11.9)	40.2 (11.4)	42.2 (12.9)
Sex, *n* (%)			
Male	4 (25.0)	4 (17.4)	4 (18.2)
Teacher experience (years), mean (SD)	15.3 (11.3)	17 (10.5)	18.1 (12.3)
Schools (*n* = 37)	*n* = 10	*n* = 12	*n* = 15
Position score (SEP), mean (SD ^a^)	−0.38 (0.63)	0.34 (0.90)	0.54 (0.91)
Food policy (T1), n			
No food policy	5	3	3
Morning break policy	1	6	4
Morning break + extra policy	4	3	8
Principle, n			
Public	1	3	7
Special ^b^	9	9	8
School size, n			
Small (<150 students)	1	6	5
Medium (150–400 students)	9	6	10
Large (>400 students)	0	0	0
Location, n			
City (>100.000 citizens)	1	1	3
Small city (10.000–100.000 citizens)	5	2	8
Town (<10.000 citizens)	4	9	4

^a^ Position score social economic position (SEP) based on the zip code of the school. Mean status for the Netherlands is 0; values >0 indicate a neighborhood with more social deprivation. ^b^ Special schools contain an independent management and are based on a specific religion or educational philosophy, such as religious-, Montessori-, Steiner-, Dalton- or Jenaplan schools. * The control group had higher nutrition knowledge compared to the intervention groups (*p* < 0.05).

**Table 4:** Short- and long-term intervention effects on children’s nutrition knowledge for the total sample (*n =* 1386) *^a^*.

		Nutrition Knowledge, Score Mean (95%CI)			T0–T1		T0–T2	
Group	n	T0	T1	T2	Change	β (95% CI) *^b^*	Change	β (95% CI) *^b^*
Control	409	3.13 (3.00, 3.27)	3.17 (3.00, 3.34)	3.37 (3.24, 3.50)	0.04	ref	0.24	ref
FV	444	2.90 (2.77, 3.03)	3.03 (2.87, 3.19)	3.22 (3.10, 3.34)	0.13	0.10 (−0.05, 0.25)	0.32	0.08 (−0.05, 0.22)
FV + Ed	533	2.92 (2.81, 3.04)	3.18 (3.03, 3.32)	3.31 (3.20, 3.42)	0.26	0.22 (0.08, 0.36) **	0.39	0.16 (0.03, 0.29) *

*^a^* = Analyses are adjusted for children’s age and sex. *^b^* = β indicates the difference in nutrition knowledge over time in the intervention group compared with the differences in nutrition knowledge over time in the control group. * *p* < 0.05, ** *p* < 0.01.

**Table 5:** Short- and long-term intervention effects on children’s FV intake (gram/day/student) for the total sample (*n =* 1386) *^a^*.

		Total FV Intake, g/Day/Student Mean (95%CI)			T0–T1		T0–T2	
Group	n	T0	T1	T2	Change	β (95% CI) *^b^*	Change	β (95% CI) *^b^*
Control	409	323 (284, 362)	316 (276, 356)	283 (251, 316)	−7	ref	−40	ref
FV	444	328 (291, 365)	350 (313, 388)	330 (299, 361)	22	29.7 (−24.8, 84.2)	2	41.6 (−18.2, 101.3)
FV + Ed	533	330 (296, 363)	365 (331, 399)	296 (268, 325)	35	43.2 (−8.9, 95.3)	−34	6.6 (−50.5, 63.8)

*^a^* = Analyses are adjusted for children’s age and sex. *^b^* = β indicates the difference in FV intake (gram/day/student) over time in the intervention group compared with the differences in FV intake over time in the control group.
